# Supplementary material for: The role and robustness of the Gini coefficient as an unbiased tool for the selection of Gini genes for normalising expression profiling data
Source: Sci Rep. 2019 Nov 29;9:17960. doi: 10.1038/s41598-019-54288-7 (PMC6884504; doi:10.1038/s41598-019-54288-7)
Supplement: Supplementary file 1 — Supplementary Information [file 41598_2019_54288_MOESM1_ESM.docx]

# Supplementary information

# The role and robustness of the Gini coefficient as an unbiased tool for the selection of Gini genes for normalising expression profiling data

^1^Marina Wright Muelas*, ^1^Farah Mughal, ^2,3^Steve O’Hagan, ^3,4,*^Philip J. Day & ^1,5*^Douglas B. Kell

^1^Department of Biochemistry, Institute of Integrative Biology, Faculty of Health and Life Sciences, University of Liverpool, Crown Street, Liverpool, L69 7ZB, UK

^2^School of Chemistry, ^3^The Manchester Institute of Biotechnology, 131, Princess St, Manchester M1 7DN, UK. ^4^Faculty of Biology, Medicine and Health, The University of Manchester M13 9PL, UK.

^5^Novo Nordisk Foundation Centre for Biosustainability, Technical University of Denmark, 10 Building 220, Kemitorvet, 2800 Kgs. Lyngby, Denmark

Emails in order of authorship: [m.wright-muelas@liverpool.ac.uk](mailto:m.wright-muelas@liverpool.ac.uk), [Farah.Mughal@liverpool.ac.uk](mailto:Farah.Mughal@liverpool.ac.uk), [SOhagan@manchester.ac.uk](mailto:SOhagan@manchester.ac.uk) , [Philip.J.Day@manchester.ac.uk](mailto:Philip.J.Day@manchester.ac.uk) , [dbk@liv.ac.uk](mailto:dbk@liv.ac.uk)

*corresponding authors: [m.wright-muelas@liverpool.ac.uk](mailto:m.wright-muelas@liverpool.ac.uk), [Philip.J.Day@manchester.ac.uk](mailto:Philip.J.Day@manchester.ac.uk), and [dbk@liv.ac.uk](mailto:dbk@liv.ac.uk)

## Supplementary Figures


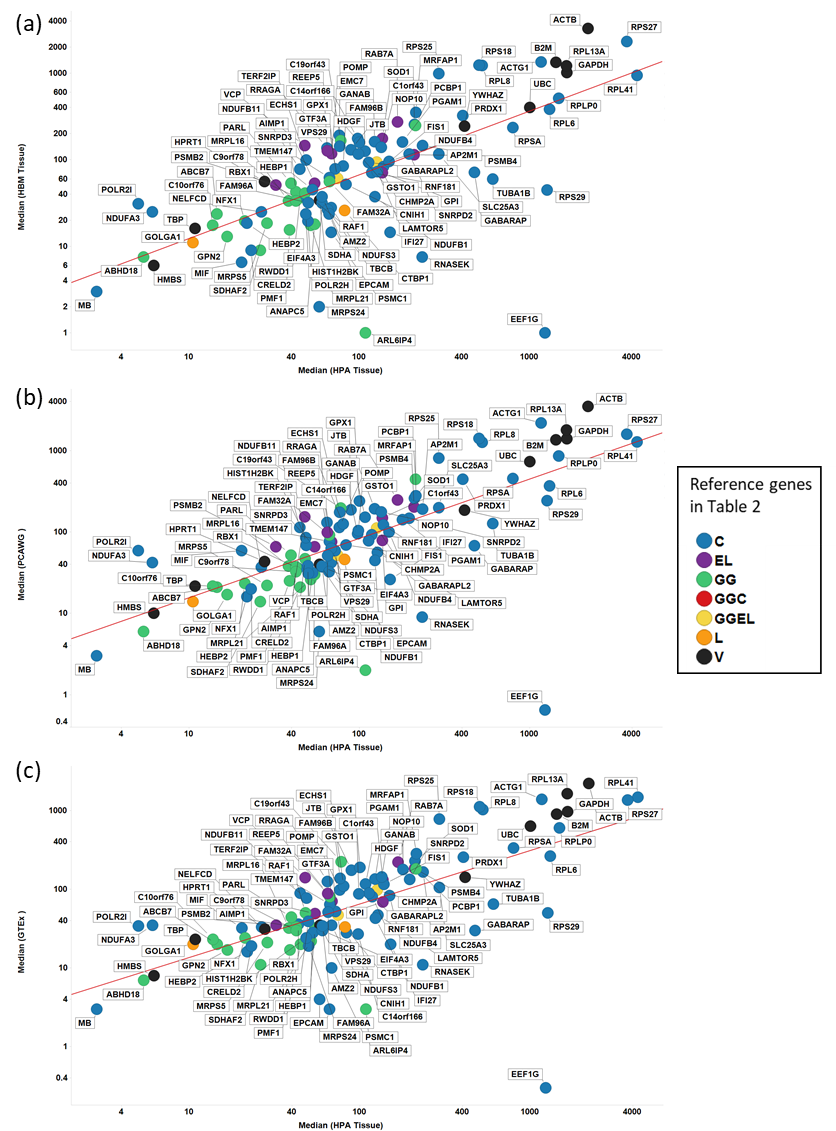


**Supplementary Figure S1. Comparison of median expression levels of proposed reference genes between tissue datasets.** A. HBM vs HPA tissue datasets. Line of best linear fit (in log space) shown is log_10_y = 0.35 + (0.74 log_10_ (x)) (r^2^=0.472). B. PCAWG vs HPA tissue dataset. Line of best linear fit (in log space) shown is log_10_y = 0.46 + (0.73 log_10_ (x)) (r^2^=0.500). C. GTEx vs HPA Tissue. Line of best linear fit (in log space) shown is log_10_y = 0.45 + (0.68 log_10_(x)) (r^2^=0.429). Colour coding: blue, Caracausi reference genes; purple, Eisenberg & Levanon; green, GeneGini; yellow, both GeneGini and Eisenberg and Levanon; orange, Lee; black, Vandesompele.

**
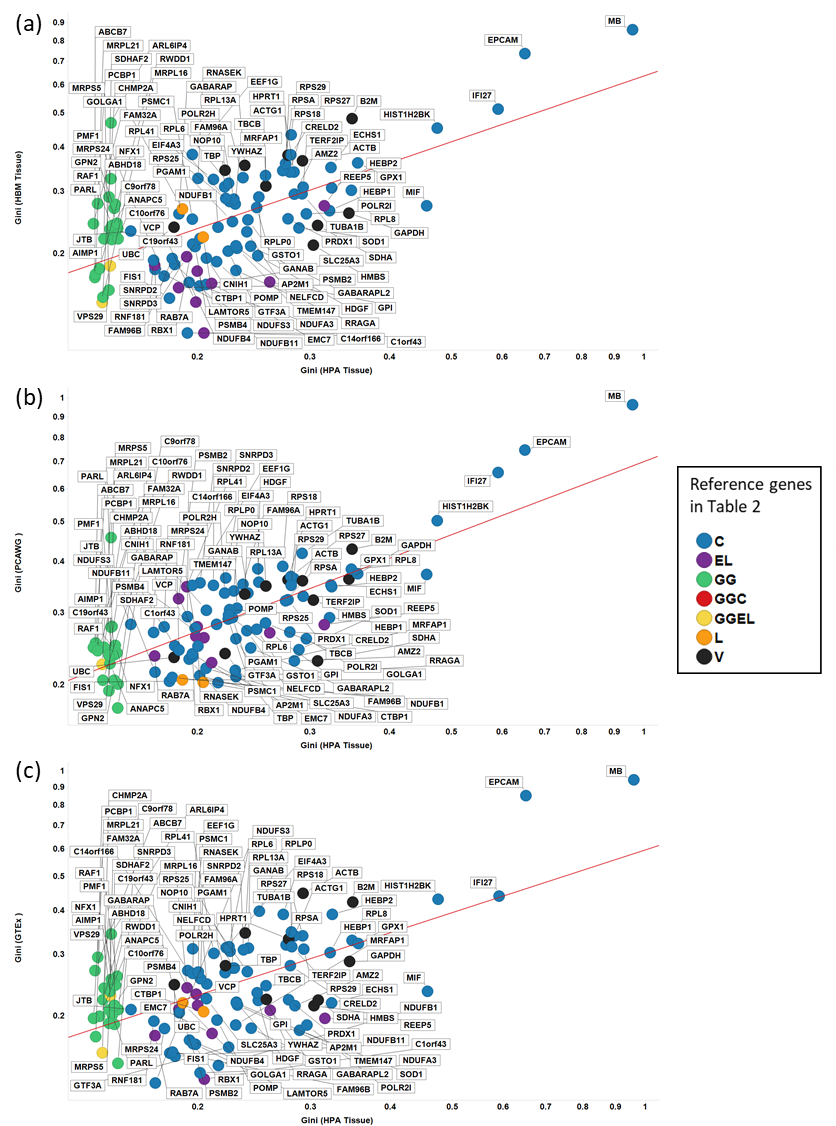
**

**Supplementary Figure S2. Comparison of Gini coefficient of proposed reference genes between tissue datasets.** A. HBM vs HPA tissue datasets. Line of best linear fit (in log space) shown is log10y = -0.20 + (0.62 log_10_(x)) (r^2^=0.392). B. PCAWG vs HPA tissue dataset. Line of best linear fit (in log space) shown is log_10_y = -0.15 + (0.59 log_10_(x)) (r^2^=0.560). C. GTEx vs HPA Tissue. Line of best linear fit (in log space) shown is log_10_y = 0.22 + (0.59 log_10_(x)) (r^2^=0.388). Colour coding as in Fig. S1.

**
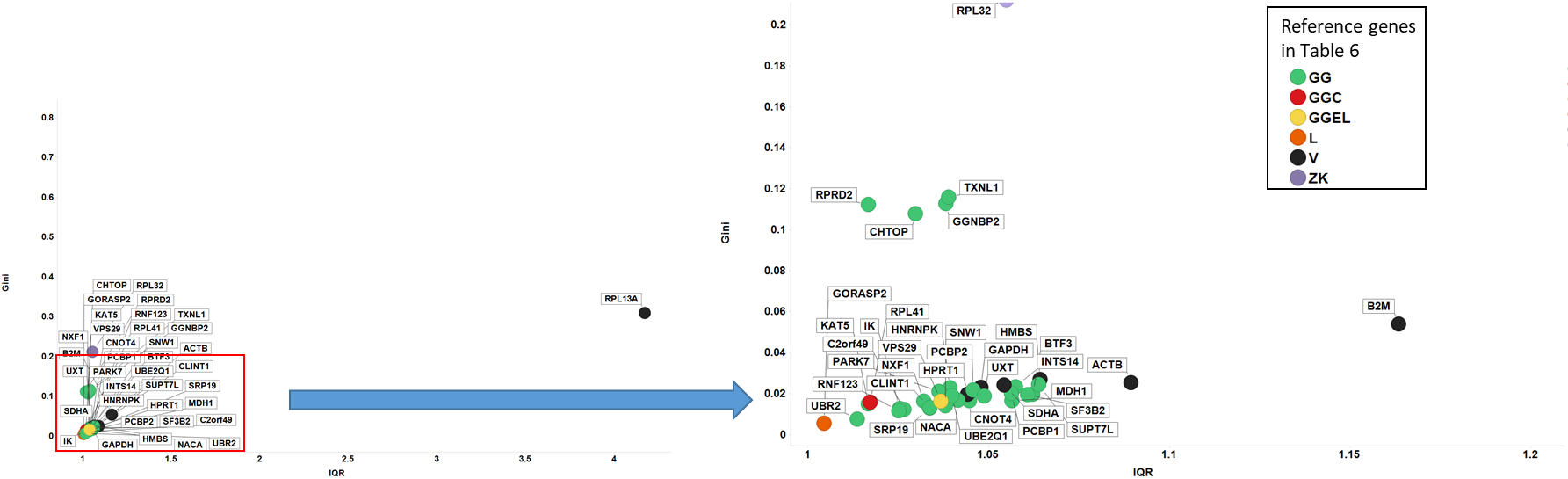
**

**Supplementary Figure S3. Robustness of the Gini coefficient assessed experimentally by RT-qPCR using a small subset of proposed reference genes illustrated with Gini coefficient vs IQR.** Left panel shows all 40 genes in Table 6, with right panel showing genes with a GC < 0.2. Colour coding: green, GeneGini reference genes; red, both GeneGini and Caracausi reference genes; yellow, GeneGini and Eisenberg and Levanon; orange, Lee, yellow; black, Vandesompele; purple, Zhang and Kriegova.

## Legends to Supplementary Tables

Supplementary Table S1. Descriptive statistics 115 common genes across cell-line datasets. S/A/O refers to SLC, ABC or Other respectively.

Supplementary Table S2. Descriptive statistics and UniProt names and IDs of proposed stable reference genes from Table 1 in tissue datasets. S/A/O refers to SLC, ABC or Other respectively.

Supplementary Table S3. Descriptive statistics of common and unique genes across tissue data sets with a GC ≤ 0.2. S/A/O refers to SLC, ABC or Other respectively.

Supplementary Table S4. Data underpinning UpSetR ^1^ plot in Figure 10 showing genes with a GC <0.2 that are variously shared and unique across the PCAWG, HBM, GTEX and HPA tissue data sets.

Supplementary Table S5. Raw expression data for candidate reference genes in human cell lines by RT-qPCR.

Supplementary Table S6. Descriptive statistics and Gini coefficient data for candidate reference genes in human cell lines by RT-qPCR.

Supplementary Table S7. Extracted analyses of cell-line RNA-Seq data sets referenced in Table 2. S/A/O refers to SLC, ABC or Other respectively.

Supplementary Table S8. Extracted analyses of tissue RNA-Seq data sets referenced in Table 2. S/A/O refers to SLC, ABC or Other respectively.

Supplementary Table S9. Primer and amplicon sequences of candidate reference genes used to assess expression stability experimentally by RT-qPCR. Included are the Gini coefficient and median expression level as found in the HPA cell-line data set. S/A/O refers to SLC, ABC or Other respectively.

## Legend to Supplementary File

Supplementary File 1. KNIME workflow ^2-4^ that we wrote to calculate descriptive statistics, including the GC, of gene expression across cell lines to assess of expression stability of candidate reference genes by RT-qPCR.

## References

1 Conway, J. R., Lex, A. & Gehlenborg, N. UpSetR: an R package for the visualization of intersecting sets and their properties. *Bioinformatics* **33**, 2938-2940, doi:10.1093/bioinformatics/btx364 (2017).

2 Mazanetz, M. P., Marmon, R. J., Reisser, C. B. T. & Morao, I. Drug discovery applications for KNIME: an open source data mining platform. *Curr Top Med Chem* **12**, 1965-1979, doi:10.2174/1568026611212180004 (2012).

3 Fillbrunn, A. *et al.* KNIME for reproducible cross-domain analysis of life science data. *J Biotechnol*, doi:10.1016/j.jbiotec.2017.07.028 (2017).

4 O'Hagan, S. & Kell, D. B. The KNIME workflow environment and its applications in Genetic Programming and machine learning. *Genetic Progr Evol Mach* **16**, 387-391, doi:10.1007/s10710-015-9247-3 (2015).
